# Febru’y Report of Deaths and Interments in Baltimore

**Published:** 1881-04

**Authors:** 


### FEBRU'Y REPORT OF DEATHS AND INTERMENTS IN BALTIMORE.

| CAUSE OF DEATH. | No. |
|---|---|
| Abscess | 2 |
| Aneurism of aorta | 1 |
| Angina pectoris | 2 |
| Apoplexy | 18 |
| Asthma | 4 |
| Asthenia | 4 |
| Bright's disease | 12 |
| Burns | 5 |
| Bronchial catarrh | 1 |
| Capillary bronchitis | 7 |
| Catarrh of lungs | 1 |
| Cancer, bowels | 1 |
| " breast | 5 |
| " liver | 1 |
| " ventricular | 1 |
| " uterus | 1 |
| " ovary | 1 |
| " stomach | 3 |
| Child birth | 1 |
| Cirrhosis of liver | 2 |
| Congestion of brain | 8 |
| " lungs | 4 |
| Consumption of Lungs | 102 |
| Convulsions | 36 |
| " puerperal | 2 |
| Croup | 13 |
| Cyanosis | 3 |
| Cirrhosis | 1 |
| Dentition | 5 |
| Delirium tremens | 1 |
| Diabetes | 2 |
| Diarrhœa | 4 |
| Diphtheria | 20 |
| Disease of heart | 28 |
| " spine | 1 |
| Dropsy, (gen'l) | 4 |
| Drowned | 2 |
| Dysentery | 3 |
| Epilepsy | 1 |
| Endocarditis | 1 |
| Erysipelas | 3 |
| Effusion of brain | 1 |
| Exhaustion | 1 |

| CAUSE OF DEATH. | No. |
|---|---|
| Fever, malarial | 3 |
| " puerperal | 2 |
| " catarrhal | 1 |
| " intermittent | 1 |
| " remittent | 2 |
| " rheumatic | 2 |
| " scarlet | 20 |
| " typhoid | 5 |
| " typho-malarial | 1 |
| Fracture, jaw | 1 |
| " skull | 1 |
| " thigh | 1 |
| " vertebra | 1 |
| Gangrene of leg | 1 |
| " lungs | 2 |
| Gastro enteritis | 3 |
| Gastric catarrh | 1 |
| Hernia, strangulated | 1 |
| Hemorrhage, bowels | 1 |
| " lungs | 1 |
| " navel | 2 |
| " brain | 1 |
| Hydrocephalus | 6 |
| Hydrothorax | 1 |
| Inanition | 8 |
| Inflammation of bladder | 1 |
| " bowels | 4 |
| " stomach | 1 |
| " brain | 3 |
| " kidneys | 3 |
| " larynx | 1 |
| " liver | 1 |
| " pericard'm. | 1 |
| " peritoneum | 2 |
| " pleura | 2 |
| " bronchi | 7 |
| Intussusception | 2 |
| Intemperance | 3 |
| Jaundice | 1 |
| Malaria | 1 |
| Malformation | 1 |
| Marasmus | 10 |
| Measles | 3 |

| CAUSE OF DEATH. | No. |
|---|---|
| Mercurial Dyscrasia | 1 |
| Meningitis | 14 |
| " Tubercular | 8 |
| " cerebro spinal | 4 |
| Murder | 2 |
| Nervous prostration | 2 |
| Old Age | 19 |
| Oedema of lungs | 1 |
| Opium habit | 1 |
| Paralysis | 10 |
| Pneumonia | 45 |
| " typhoid | 3 |
| " pleuro | 4 |
| " broncho | 2 |
| Poison | 1 |
| Premature birth | 9 |
| Protracted labor | 3 |
| Pulmonary embolism | 1 |
| Ricketts | 1 |
| Rheumatism | 4 |
| Results of parturation | 2 |
| Scrofula | 7 |
| Softening of brain | 1 |
| Septicæmia | 1 |
| Stricture of intestines | 1 |
| Stomatitis materna | 1 |
| Starvation | 1 |
| Suicide | 1 |
| Syphilis | 7 |
| Tabes Mesenterica | 1 |
| Tetanus | 1 |
| Tuberculous mesenterica | 1 |
| Toxæmia | 1 |
| Tumor, chest | 1 |
| " ovarian | 2 |
| Uræmia | 3 |
| Unknown Infantile | 3 |
| Whooping Cough | 4 |
| Wounds | 5 |
| Total | 614 |

THE DEATHS REGISTERED IN THE MONTH INCLUDE

| | |
|---|---|
| Under 1 year | 136 |
| Between 1 and 2 years | 56 |
| " 2 and 5 years | 43 |
| Under 5 years | 235 |
| Between 5 and 10 years | 37 |
| " 10 and 15 " | 15 |
| " 15 and 20 " | 18 |
| Between 20 and 30 " | 64 |
| " 30 and 40 " | 50 |
| " 40 and 50 " | 35 |
| " 50 and 60 " | 48 |
| " 60 and 70 " | 54 |
| " 70 and 80 " | 42 |
| " 80 and 90 " | 10 |
| " 90 and 110 " | 6 |

NATIVITY, ETC.

| | |
|---|---|
| United States—White | 344 |
| " Colored | 181 |
| Foreign | 89 |
| Total for the month | 614 |
| Still Birth | 60 |

Among the decedents were 63 above the age of 70 years, whose combined ages were 4,935 years, averaging 78 years 4 months.

Estimated Population, 393,796. white, 338,384. Colored, 55,412. Annual Death Rate during the month, whole population, per 1,000—18.70. White, 15.37. Colored, 39.49.
Births reported, 587.

By Order: JAMES A. STEUART, M. D., Commissioner of Health and Registrar

A. R. CARTER, Secretary.

U. S. SIGNAL OFFICE, BALTIMORE, MD., March 1, 1881.

### METEOROLOGICAL SUMMARY FOR THE MONTH OF FEBRUARY, 1881.

### PRESSURE.

| | | |
|---|---|---|
| Mean Monthly Barometer, | 30.220 inches. | |
| " " " for past 10 years, | 30.100 " | |
| Highest Barometer, | 30.869 " | on the 7th. |
| Lowest " | 29.409 " | on the 12th. |
| Monthly Range of Barometer, | 1.460 " | |

### TEMPERATURE.

Mean Monthly Thermometer, - - - - - 34.8 degrees.
" " " for past 10 years - 37.2 "
Highest Thermometer, - 64 degrees on 12th. Monthly Range – 60 degrees.
Lowest " - 4 degrees on 2d. Mean relative humidity, 66.7 "

### WIND, ETC.

Prevailing direction of wind, northwest.
Highest velocity of wind, 28 miles per hour.
Number of days on which rain fell, 13.
Total amount of rainfall, 5.68 inches.
Total movement of wind, 4,332 miles.
Total rainfall, 6.68 Inches.

ROBERT SEYBOTH, in charge.

